# Electrical Accidents to Domestic Animals

**Published:** 1891-12

**Authors:** James A. Waugh

**Affiliations:** Alleghany, Pa.


### ELECTRICAL ACCIDENTS TO DOMESTIC ANIMALS.

By James A. Waugh, V. S.
Alleghany, Pa.

The application and utilization of electricity as a motor-power for street railway coaches, and the use of cheap or badly constructed or defective arrangements in road-beds, prove sources of occasional serious accidental electrical injuries to horses that are driven over these car-tracks, and come in contact with rails, which have become sur-charged with an excess of electricity due to a settling or sinking of the road-bed and breaking of the underground wires, which cause breaks in the electric circuits.

Horses are often injured by attempting to cross from one set of tracks to another while cars are approaching in opposite directions, and especially when approaching river-bridges. The horse happens to step with one fore and one hind foot, or both fore and hind feet at the same time on the inside rails of the double tracks, and the metallic shoes on the horses feet act as conductors which transmit the electricity to the animal and causes it to rear or spring several feet up into the air, and then fall prostrate on the street; while other cases are shocked so severely that they fall prostrate and remain paralyzed. The character of the injury is in proportion to the amount of electricity received, intensified by violent contact with the street. The shock may be so severe as to cause instant death or only partial paralysis with almost complete prostration for a few hours or several days, weeks or months. The pulse is slow, feeble and irregular; nostrils dilated, respiration slow and laborious; temperature slightly elevated; pupils dilated or contracted, the eyes present a peculiar and unnatural expression, while sometimes strabismus is present, and at other times the eyelids are paralyzed: prespiration rather free in the early stages; mastication and deglutition impaired, some patients will require an hour or more to drink a pail of water. The head is sometimes held almost in a line with the neck or twisted on one side with the eye pointing toward the ground; one ear may be held erect while the other is lopped and paralyzed. Locomotion is seriously impaired and the animal stands with its feet wide apart as if trying to brace and steady itself, and when moved the feet are raised only a slight distance from the ground; sometimes the patient is unable to assume a standing position, while others walk and act somewhat like human beings affected with *locomotor ataxia*, staggers from side to side, if hurried falls down and turns somersaults in all directions. The animal appears much frightened and nervous and there may be a well-marked quivering of certain sets of muscles for several days or weeks after the accident. The functions of the digestive and urinary organs are somewhat impaired in the early stages but soon regain their normal condition. The nerve cells are seriously injured and the functions of the nervous system is impaired and sometimes permanently damaged.

I have not yet had an opportunity for post-mortem examinations on this subject, but Dr. Jackson informed me that Dr. Jennings and himself had found well-marked congestion of the mesenteric glands in a horse which had been killed by an electric shock received on street-car tracks, and heart greatly hypertrophied, which probably accounts for the sudden death. Elevated wires are sometimes displaced and prove fatal to horses which come in contact with them.

Treatment consists in hypodermic injections of strychnia, atropine, digitaline, and administration of stimulents and restoratives, either in drenches or enemas. It is sometimes necessary to secure and confine the patient in slings for several weeks or months, but mild cases usually do nicely in comfortable box-stalls. Feed and water from high mangers. Tonics and alteratives are beneficial during convalescence, which is generally tardy and unsatisfactory. The owners usually desire post-mortem examinations of horses killed by electricity; while horses severely shocked and injured are generally treated for some time in order that damages may be legally recovered from the traction company. A few cases may be cited to illustrate the above remarks. I have had a case under treatment and observation for about four months and it is not yet well enough to walk out of the stable; although the companies veterinarian diagnosed it as a simple case of pleurisy which would recover in a few days, while two of the ablest veterinarians in the city were called in as evidence and confirmed my diagnosis —electric shock.

Dr. J. C. McNeil, kindly showed me a horse which had been shocked about a year ago and was driven three miles to the country, the patient suffered severely and was unable to control his actions and it was found necessary to confine him in slings for a period of twenty-one weeks, This horse is not yet fit for any work.

I saw a large heavy draft horse that was shocked early last winter, and the owner finally became discouraged and gave the horse away to a farmer. There is a great variety of mild forms of electrical injuries which appear to cause a reduced or increased function of certain sets of muscles of locomotion, especially the abductors, flexors, and extensors of the posterior extremities.

